# The absence of dystrophin brain isoform expression in healthy human heart ventricles explains the pathogenesis of 5' X-linked dilated cardiomyopathy

**DOI:** 10.1186/1471-2350-13-20

**Published:** 2012-03-28

**Authors:** Marcella Neri, Emanuele Valli, Giovanna Alfano, Matteo Bovolenta, Pietro Spitali, Claudio Rapezzi, Francesco Muntoni, Sandro Banfi, Giovanni Perini, Francesca Gualandi, Alessandra Ferlini

**Affiliations:** 1 Department of Experimental Diagnostic Medicine, Section of Medical Genetics, University of Ferrara, Ferrara, Italy; 2 Department of Biology, University of Bologna, Bologna, Italy; 3 Telethon Institute of Genetics and Medicine, Naples, Italy; 4 Institute of Cardiology, University of Bologna and S. Orsola Malpighi Hospital, Bologna, Italy; 5 Dubowitz Neuromuscular Centre, UCL Institute of Child Health, London, UK

## Abstract

**Background:**

In X-linked dilated cardiomyopathy due to *dystrophin *mutations which abolish the expression of the M isoform (5'-XLDC), the skeletal muscle is spared through the up-regulation of the Brain (B) isoform, a compensatory mechanism that does not appear to occur in the heart of affected individuals.

**Methods:**

We quantitatively studied the expression topography of both B and M isoforms in various human heart regions through *in-situ *RNA hybridization, Reverse-Transcriptase and Real-Time PCR experiments. We also investigated the methylation profile of the B promoter region in the heart and quantified the B isoform up regulation in the skeletal muscle of two 5'-XLDC patients.

**Results:**

Unlike the M isoform, consistently detectable in all the heart regions, the B isoform was selectively expressed in atrial cardiomyocytes, but absent in ventricles and in conduction system structures. Although the level of B isoform messenger in the skeletal muscle of 5'-XLDC patients was lower that of the M messenger present in control muscle, it seems sufficient to avoid an overt muscle pathology. This result is consistent with the protein level in XLDC patients muscles we previously quantified. Methylation studies revealed that the B promoter shows an overall low level of methylation at the CG dinucleotides in both atria and ventricles, suggesting a methylation-independent regulation of the B promoter activity.

**Conclusions:**

The ventricular dilatation seen in 5'-XLDC patients appears to be functionally related to loss of the M isoform, the only isoform transcribed in human ventricles; in contrast, the B isoform is well expressed in heart but confined to the atria. Since the B isoform can functionally replace the M isoform in the skeletal muscle, its expression in the heart could potentially exert the same rescue function. Methylation status does not seem to play a role in the differential B promoter activity in atria and ventricles, which may be governed by other regulatory mechanisms. If these mechanisms could be deduced, de-silencing of the B isoform may represent a therapeutic strategy in 5'-XLDC patients.

## Background

The *dystrophin *gene [GenBank at the NCBI: NG_012232.1] encodes for a high molecular weight cytoskeletal protein primarily expressed in the skeletal and cardiac muscles, and to a lesser extent in smooth muscles and brain. The large *dystrophin *gene generates multiple transcripts, resulting in several isoforms, which are produced by both alternative splicing events and tissue-specific promoter usage. Seven promoters are known to initiate dystrophin transcription; three of these, the brain (B), muscle (M) and Purkinje (P) promoters are clustered at the 5' end of the gene and generate full length 14-Kb transcripts with an unique first exon spliced to a shared set of 78 exons [1].

The M transcript represents the prevalent dystrophin isoform in both skeletal and cardiac muscles, whereas the expression patterns of the other full-length dystrophin isoforms (B and P) have been less well characterized in these tissues. The B isoform has been inconsistently detected in both the whole heart and skeletal muscle of adult human individuals [2-6]. In foetal human tissues, the B transcript has been identified in both the heart and in the skeletal muscle, although its transcription appears to be activated at different developmental stages [6,7]. The dystrophin P isoform, on the other hand, is undetectable in human foetal and adult hearts and in foetal skeletal muscle [5-7], being confined to adult skeletal muscle [5,6].

Dystrophin deficiency causes different allelic clinical phenotypes, as Duchenne (DMD, OMIM 310200) and Becker (BMD, OMIM 300376) muscular dystrophies; the skeletal muscles are mainly affected but with the progression of the disease there is also an overt cardiomyopathy. In contrast, X-Linked Dilated Cardiomyopathy (XLDC, OMIM 302045) due to *dystrophin *gene mutations is characterized by selective cardiac disease with no significant skeletal muscle symptoms [1]. Nonetheless, elevated serum creatine kinase (CK) values in this condition do suggest a subclinical skeletal muscle involvement.

A classification of XLDC cases based on both molecular and clinical features has been proposed, distinguishing milder forms with delayed presentation associated with mutations in the spectrin-like dystrophin domain (3'-XLDC) from the more severe early-onset forms caused by 5' *dystrophin *gene mutations (5'-XLDC) [8,9]. Notably, a significant group of 5'-XLDC mutations specifically affects the expression of the M isoform [10-15] and result in a common transcriptional pattern characterized by the up-regulation of dystrophin B (predominantly) and P isoforms in the skeletal muscle of patients but not in the heart [7,10-12,15,16]. This transcriptional behaviour has been suggested as the pathogenic basis behind the exclusive cardiac involvement in 5'-XLDC; in fact, while the cardiac tissue suffers from M isoform deficiency, the skeletal muscle is spared as a result of the compensatory up-regulation of B and P transcripts. This view has been supported by the experimental observation that the *dystrophin *muscle enhancer 1 (DME1), which is involved in the *in vitro *up-regulation of B and P promoters, is active in skeletal but not in cardiac muscle-derived cell lines [15]. The DME1 selective activation of P promoter in skeletal muscle has also been confirmed in a transgenic mouse model *in vivo *[17], thereby implicating the inability of DME1 to up-regulate B and P isoform in the heart in the cardiac pathology, at least in a murine model. Furthermore, the *DOT1L *gene, which acts as a disruptor of telomeric silencing, has recently been ascribed a regulatory role in *dystrophin *expression in both the *mdx *mouse and dilated cardiomyopathy (DCM) patient tissues [18]. This observation directly implicates methylation regulation in *dystrophin *expression, adding novel clues into the pathogenesis of XLDC.

In order to shed more light on the mechanisms behind the lack of compensatory up-regulation of the B isoform in the 5'-XLDC heart, we investigated the transcription pattern of the B isoform in normal human heart compartments and in the whole human heart. We also studied the methylation status of the B promoter in heart compartments, looking in particular for regional differences that could account for the variation observed in isoform expression. Furthermore, we quantified the B isoform up-regulation in the skeletal muscle of two 5' XLDC patients lacking the M isoform [7,10] and compared it to the normal level of dystrophin M transcript in control skeletal muscle. We showed that, in contrast to the M isoform, which is uniformly detectable in all the heart regions, the B isoform is selectively expressed in atrial cardiomyocytes, but absent in the ventricles and in conduction system structures. Methylation studies of the B promoter genomic region in the heart revealed a uniformly homogeneous low level of methylation of CG dinucleotides. No differences in the methylation status were found between the atria and ventricles, thereby suggesting that exists a methylation-independent regulation of the B promoter activity. Thus, we speculate that the B promoter activity might be driven by other regulatory mechanisms which need to be elucidated in order to find strategies able to activate the B isoform transcription; this could be of therapeutic value in 5'XLDC patients.

## Results

### RT-PCR analysis

Reverse-Transcriptase (RT) PCR amplification of *dystrophin *B and M isoforms revealed different expression patterns on RNA from both different human cardiac areas and total human cardiac tissue; the M isoform was found to be uniformly expressed in all the areas examined as was the housekeeping gene actin. In contrast, the expected PCR product corresponding to the B isoform was detectable only in atrial samples; sequence analysis confirmed this 514-bp PCR product as the expected B-specific exon1-exon 6 dystrophin fragment (data not shown).

**Figure 1:**
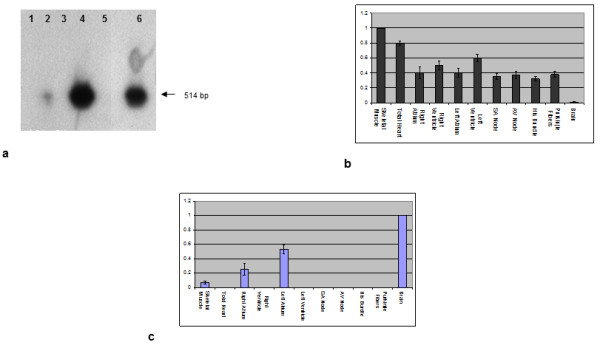
**a. Hybridization of *dystrophin *B isoform RT-PCR (oligonucleotides within B exons 1 and 6; expected product: 514 bp) with an internal oligo probe (within exon 2) that confirmed its expression in both Atria (lanes 2 and 4) and in the control brain (lane 6), and its absence in the Ventricles and Total Heart**. **b **Levels of *dystrophin *M transcript expression (2-ΔΔCT values) using the skeletal muscle as internal calibrator and *actin *as reference gene in: Skeletal Muscle, Total heart, Right Atrium, Right Ventricle, Left Atrium, Left Ventricle, Sinoatrial (SA) Node, Atrioventricular (AV) Node, Bundle of His and Purkinje Fibres. The M isoform was expressed in all the samples studied, featuring the lowest score in Brain. **c **Levels of *dystrophin *B transcript expression (2-ΔΔCT values) using the brain as internal calibrator and *actin *as reference gene in the same samples. In both the ventricles the B amplification threshold cycle (CT) was undetermined, as well as in the total heart and in the conduction system structures. In the atria the score obtained was higher than that in skeletal muscle, in particular in the left atrium.

RT-PCR product hybridization with an oligo probe specific for dystrophin exon 2 ruled out the occurrence of a faint B isoform expression. Indeed this hybridization pattern confirmed that the atrial structures represent the only site of expression of the dystrophin B transcript in the heart, showing a prevalence in the left atrium. Neither the ventricles nor total human cardiac tissue showed the presence of any hybridization signal (Figure 1a).

### Real-time PCR analysis of B and M isoforms

Quantification of both B and M *dystrophin *transcripts was performed on commercial human RNA (Ambion^®^, Analytical Biological Services (ABS) Inc.) from total heart, atria, ventricles and conduction system structures (CCS); commercial RNA (Ambion^®^, Analytical Biological Services (ABS) Inc.) from human skeletal muscle and brain were used as controls. Expression of the M isoform was revealed in all samples examined, with the highest score being found in the skeletal muscle (acting as a calibrator), the lowest in the brain, and intermediate values in total heart, atria and ventricles (Figure 1b). The B isoform was detected at very low levels in the control skeletal muscle and showed the highest level in the brain (acting as a calibrator). Interestingly, B isoform expression values were not uniform in cardiac areas; in both ventricles and in total heart the B amplification threshold cycle (CT) was undetermined in separate experiments with different amounts of cDNA, thereby confirming the findings observed in RT-PCR experiments. In the atria, particularly the left, the level of expression was higher than in skeletal muscle. We postulate that the failure to detect the B messenger in the human cardiac tissue as a whole could be due to the prevalent ventricular origin of the commercial RNA used (Ambion^®^, Analytical Biological Services (ABS) Inc) (Figure 1c).

In the CCS compartments (sinoatrial and atrioventricular nodes, bundle of His and Purkinje fibres) the M isoform was expressed at similar levels to actin compared to the skeletal muscle as an internal calibrator (Figure 1b). In repeated experiments with increasing amounts of cDNA on the same samples, the B amplification CT was undetermined (Figure 1b), thereby implicating atrial cardiomyocytes as the elective site of expression of the cardiac B messenger.

**Figure 2:**
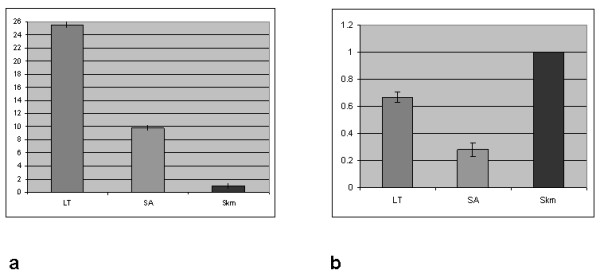
**a. Levels of *dystrophin *B transcript expression (2-ΔΔCT values) in the skeletal muscle of two 5'XLDC patients (LT and SA), as compared to a control muscle (calibrator), using *actin *as reference gene**. B upregulation resulted in 25- and 9-fold transcript levels in LT and SA, respectively. **b **Levels of *dystrophin *B transcript (2-ΔΔCT values) in the skeletal muscle of the same 5'XLDC patients, as compared to the level of *dystrophin *M transcript in a skeletal muscle control using *actin *as reference gene. The ratio was 0.67 and 0.28 in patients LT and SA, respectively.

In the skeletal muscle of two unrelated 5' XLDC patients (LT and SA, previously described [10,14]) the M amplification CT was undetermined, as expected due to their particular genomic mutation (data not shown). The B isoform expression values confirmed the up-regulation previously detected in RT-PCR experiments [10,14]. Application of the equation 2^-ΔΔCT ^to the expression of B isoform in patient skeletal muscle, as compared to a control skeletal muscle, yielded results of 25 and 9 in patients LT and SA, respectively, using actin as the reference gene (Figure 2a).

We compared the level of the B *dystrophin *transcript in the skeletal muscle of these patients with the level of total *dystrophin *transcript in a normal muscle (almost exclusively driven by the M promoter). In both patients the *dystrophin *messenger was found to be quantitatively reduced with respect to healthy control muscle (2^-ΔΔCT ^0.67 and 0.28 in LT and SA, respectively) (Figure 2b). Nevertheless, the amount of the B *dystrophin *transcript is still sufficient to guarantee protein levels (57% in LT and 29% in SA) able to avert muscular dystrophy, as we have previously reported [10].

### In-situ hybridization

**Figure 3:**
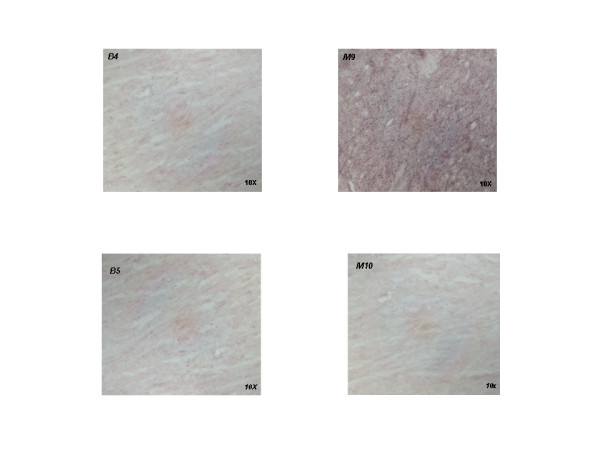
**Non-radioactive in-situ RNA hybridization on cryosections obtained from the left ventricle of an explanted human heart**. Isoform-specific RNA probes recognized the 5' UTR region and first exon of B and M isoforms. The M antisense probe (**M9 top right**) displayed an intense signal of hybridization whereas the B antisense probe failed to hybridize (**B4 top left**). Both the M (**M10 bottom right**) and B sense (**B5 bottom left**) probes failed to hybridize.

Non-radioactive in-situ hybridization experiments were carried out with isoform-specific riboprobes on a human left ventricle sample; whereas the M probe displayed an intense hybridization signal, the B probe failed to hybridize, thereby confirming the lack of B expression in this heart compartment (Figure 3). These data confirm that the B isoform has a topographically specific expression in the different heart compartments and further support the results of Reverse-Transcriptase and Real-Time PCR experiments.

### Methylation analysis

**Figure 4:**
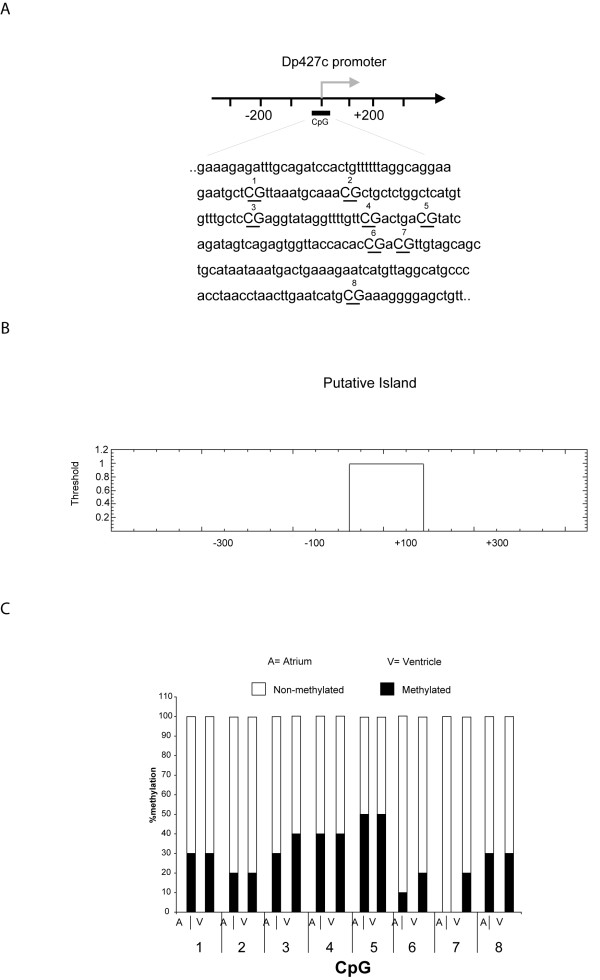
**Methylation status of the B promoter CpG islands in heart ventricle and atrium**. A) Schematic representation of the B isoform (Dp427c) promoter. Grey arrow indicates the transcription start site of the Dp427c mRNA. The DNA sequence and distribution of the CG dinucleotides is shown B) Prediction of the putative CpG island in the B promoter using the Emboss CpGplot algorithm C) Percentage of methylated cytosines within the putative CpG island in DNA extracted from ventricle and atrium muscle tissue, respectively.

Analysis of the B promoter genomic region in the heart showed an overall low level of methylation of CG dinucleotides, a finding which could indicate the presence of a CpG island in that region. On the other hand, comparison of the methylation status of DNA extracted from atria and ventricles revealed no significant differences; this could be a clue that the regulation of B isoform expression in the heart compartments is driven by a mechanism other than promoter methylation (Figure 4).

## Discussion

In medical genetics, XLDC represents an interesting example of a Mendelian disease due to mutations that selectively affect different tissues/compartments. Clearly, the elucidation of the pathogenic model(s) of this rare phenotype may impact on the correction strategies aiming at rescuing dystrophin function in the heart. Although the explanation of the isolated cardiac involvement in XLDC remains controversial, many reports have indirectly suggested that it may be due to the inability of the heart to up-regulate the B-isoform to compensate for the lack of M isoform transcription, which is selectively abolished by specific mutations in the 5' region of the gene.

In order to unequivocally define the topography of B isoform distribution and its regulatory aspects in the heart, we investigated the expression of *dystrophin *isoforms M and B in different compartments of the normal human cardiac muscle. This revealed that while the M isoform is uniformly expressed in the whole heart, including cardiac atria, ventricles and conduction system structures (CCS), the transcription of the B isoform is confined to the atria and absent in both ventricles and CCS. We support this finding by Reverse-Transcriptase and Real-time PCR assays, as well as in-situ RNA hybridisation.

This is the first clear demonstration that the B transcript is absent in normal human heart ventricles, structures dedicated to contractile function and primarily affected in XLDC. Furthermore, these data support the hypothesis that the abolition by 5' *dystrophin *mutations of M transcript, which we demonstrate is the exclusive dystrophin isoform present in the heart ventricles, is the pathogenic mechanism leading to the selective cardiac involvement in patients with 5' -XLDC. In fact the skeletal muscle in these 5'-XLDC patients seems to be able to compensate for the absence of the M isoform by up-regulating B-isoform expression. The inability of the heart to benefit from the up-regulation of the B isoform is related to its peculiar expression topography. In keeping with our molecular findings, echocardiographic clues for the diagnosis of XLDC consist of the detection of ventricular non-compaction and localized inferobasal left ventricular hypokinesia/akinesia, with no sign of primary atrial involvement [19]. It therefore appears that the pathogenesis of 5' XLDC is due to the loss of the only isoform physiologically transcribed in these structures, the M isoform.

Moreover, our data attest to the existence of regional intra-cardiac differences in representation of *dystrophin *B-isoform mRNA, to our knowledge for the first time. Atrial and ventricular myocardia exhibit fundamental differences, not only in their morphology, but also in their ultrastructure and function; indeed, the ventricular myocardium functions primarily as a contractile pump, whereas atrial myocardium has prominent neuroendocrine and chronotropic regulatory functions. It has been suggested that these differences could be related to the existence of a chamber-specific transcriptome, with ventricular myocytes expressing genes to meet contractile and energetic demands and atrial myocytes possessing transcriptional activity related to signal transduction and cell-cell communication [20]. From this perspective, the selective atrial expression of the B-dystrophin isoform could be related to the role of this isoform in signalling pathways, in keeping with its primary expression in the central nervous system. In the mature brain, the B isoform is preferentially expressed in specific regional neuronal subpopulations and localizes to punctate structures, which seem to correspond to a subset of GABAergic synapses [21]; experimental evidence has suggested a role for this isoform in maintaining cellular calcium homeostasis and calcium-related intra- and inter-cellular signalling [22,23].

Atrium-specific gene expression invariably appears to be achieved through the silencing of gene expression in the ventricular compartment during development [24]. This has been demonstrated experimentally for genes *MLC2a *(Myosin Light Chain atrial), *slow MyHC3 *(Slow Myosin Heavy Chain) and *ANF *(Atrial Natriuretic Factor) [25-27]. We speculate that the differences in B-isoform expression between atria and ventricles in the adult heart could be the result of a similar silencing mechanism, which occurs during development and privileges the exclusive expression of the M isoform, due to its primary contractile function in ventricles. Considering the very high rate of ATP consumption, linked to the sarcomeric activity, in the ventricles, it is not surprising that cardiomyocytes favour the transcription (known to be a high-cost process for the cell) of only one full-length *dystrophin *messenger [8]. However, this selective transcription pattern seems to expose the ventricles to mutations affecting M isoform synthesis, including XLDC and dystrophinopathies in general.

The B promoter belongs to a family of ancient, prokaryotic-like CAAT- and TATA-less promoters, which are known to display low responsiveness to regulation through CpG island methylation by methyl-binding proteins [2,28]. Indeed, we described a low methylation level of CG dinucleotides of the B promoter and no differences of methylation between atria and ventricles, supporting that the regulation of the B isoform expression in the heart compartments is driven by a mechanism other than promoter methylation. These data, associated to the evidence that DME1 enhancer (acting in a pre-transcriptional mechanism) and DOT1 (a telomeric silencing inhibitor, which acts at post-transcriptional level) both regulate the dystrophin isoforms in the heart [18], reveal further complexity in the tissue-/compartment-specific control of *dystrophin *gene expression. It is likely that genomic regions surrounding the B and M promoters, as well as genomic regions upstream of the gene, could harbour elements that orchestrate the expression of full-length isoforms in a particular tissue in a time-specific way. In particular, locus control regions (LCR) are the DNA sequences that define a chromatin domain of an independent regulatory environment, and are characterized by a set of DNase-I hypersensitive sites (HSS), which contain binding sites for a variety of regulatory proteins. Several studies have suggested that LCRs are indispensable for appropriate execution of a developmental regulation programme [29-31]. In addition, post-transcriptional regulatory mechanisms such as those highlighted by RNA silencing (RNAsi), orchestrated by a variety of small RNAs, could play a relevant role in *dystrophin *expression modulation, as recently reported [32,33].

Although a major non-mechanical function of the B isoform seems to be supported by its atrial and central nervous system expression, the up-regulation of this isoform in the skeletal muscle of 5'-XLDC patients is clearly capable of protecting muscle from progressive muscle degeneration. This suggests that this isoform has preserved structural potential, which is not surprising, as the difference between the M and B isoforms is confined to the amino-acids encoded by exon 1 (eleven for the M isoform and three for the B isoform), leaving the actin and beta dystroglycan domains of dystrophin intact.

## Conclusions

Investigation of the distribution pattern of full-length dystrophins in cardiac muscle revealed a difference in the expression of M and B isoforms between atria and ventricles. Nonetheless, despite the identification of the various core promoters and enhancer regions, the mechanisms underlining differences in regulation of *dystrophin *transcription between cardiac and skeletal muscle are still not entirely clear. From our data it is evident that *dystrophin *transcription by the various promoters is characterized by tissue selectivity rather than tissue specificity; these promoters are active, albeit at different levels, in different tissues and are developmentally regulated. Moreover, for the first time we demonstrate the existence of regional intra-cardiac differences in the representation of *dystrophin *B isoform mRNA, allowing us to shed more light on the pathogenesis of 5' XLDC.

The selective atrial expression of B isoform and the evidence that the B isoform is able to functionally replace the M isoform in the skeletal muscle may open novel therapeutic windows for patients suffering from 5' XLDC and dystrophinopathies in general.

In this view it appears promising a drug discovery approach which aims at searching for molecules/drugs able to specifically activate B promoter in human heart ventricles and thereby restoring dystrophin synthesis.

## Methods

### Reverse-transcription PCR (RT-PCR)

B and M *dystrophin *transcripts were analysed by PCR amplification of reverse-transcribed commercial RNA (Ambion^® ^Analytical Biological Services (ABS) Inc.) of human total heart as well as of different cardiac areas, i.e. right and left atria and ventricles. Reverse transcription (RT) was performed using a high capacity cDNA reverse-transcription kit (Applied Biosystems), according to the protocol supplied. The B and M transcripts were amplified by means of an exon-specific forward oligonucleotide and a reverse primer located in exon 6 (sequences are available upon request). As housekeeping gene, we amplified the actin gene on the same samples (primer sequences are available upon request).

PCR was carried out in a reaction volume of 25 μl containing the cDNA template, 2.5 U Ex Taq polymerase (Takara), 1× Ex Taq Buffer, 1.5 mM MgCl_2_, 200 μM dNTPs, and 0.5-1 μM of each primer. Amplification conditions were as follows: initial denaturation (2 min) at 94°C, denaturation (30 s) at 94°C, annealing (30 s) at 63°C, extension for 5 cycles at 68°C (30 s), followed by denaturation at 94°C (30 s), annealing at 62°C (30 s), extension (45 s) for 30 cycles at 68°C, and final extension at 68°C (3 min). 10 μl of the PCR reaction mixture was analysed on 1.3% agarose gel. RT-PCR products of B amplification were purified from the gels, cloned into pCR II vector (Invitrogen) and sequenced with M13 and T7 primers, using a Big Dye terminator cycle sequencing kit, and analysed using ABI Prism 3130 (PE Applied Biosystems).

One aliquot of the PCR reaction mixture was transferred onto a GeneScreen Plus (NEN) membrane in SSC 10× buffer. RT-PCR products were hybridized with an internal oligo probe (exon2F: 5' TGGGTAA ATGCACA ATTT TCTAAG 3'), according to standard procedures.

### RNA isolation from 5' XLDC patient skeletal muscle biopsy samples

The genotype characterization of the two 5'-XLDC patients studied, as well as their transcription patterns of *dystrophin *isoforms in the skeletal muscle, has been previously reported. The first patient (LT) possesses a splice site mutation in the first intron of the M isoform pre-mRNA [10], while the second patient (SA) features a deletion removing the muscle promoter and first muscle exon [14].

Total RNA was isolated from frozen skeletal muscle biopsies from the patients using an RNeasy Kit (Qiagen), following the manufacturer's instructions. Before cDNA synthesis, RNA was treated with DNAse I (Roche) and checked for residual DNA contamination using 55-cycle PCR.

### Real-time PCR analysis of B and M isoforms in control heart areas and 5' XLDC patient skeletal muscle

Real-time PCR was carried out using the GeneAmp Sequence Detection System 7300 (Applied Biosystems). Commercial RNAs (Ambion^®^, Analytical Biological Services (ABS) Inc.) from the following human samples were studied: skeletal muscle, brain, total heart, right atrium, right ventricle, left atrium, left ventricle, sinoatrial node, atrioventricular node, bundle of His and Purkinje fibres. Furthermore, RNA extracted from the skeletal muscle of two 5'-XLDC patients was analysed as previously described.

Primer Express^® ^Software was used for the selection of primers and MGB probes (FAM-labelled) on the 5'UTR and first exon of B and M *dystrophin *isoforms. Real-Time assay sequences are available upon request.

Real-Time PCR was performed in triplicate in a 96-well plate; each 25 μl reaction consisted of 1× Taqman Master Mix (Applied Biosystems), 300 nM forward and reverse primers, 100 nM Taqman probes and 20 ng cDNA. For the reference gene actin (ACTB), a TaqMan Endogenous control assay was utilized (ID 4333762F Applied Biosystems) according to the protocol supplied. The PCR conditions for all analysed genes were as follows: pre-heating at 50°C for 2 min and 95°C for 10 min; and cycling, 40 cycles at 95°C for 15 s and 60°C for 1 min.

The data obtained were analysed by the comparative CT method (DDCt Method) (Applied Biosystems User Bullettin #2), which evaluates the expression of the target genes (M and B dystrophin) with respect to that of the reference gene (actin), and normalizes these values relative to a calibrator sample (control Skeletal Muscle or control Brain).

### In-situ hybridization (ISH)

Non-radioactive in-situ hybridization was performed on cryosections (18 micron thickness) obtained from the left ventricle of an explanted human heart. Two series of seriate sections were prepared on each slide, and in each ISH experiment four slides from independent series were used. Probes were obtained by genomic amplification of the full 5'UTR regions and first exons of *dystrophin *M and B isoforms (oligonucleotide sequences are available upon request). For M isoforms the amplification product was 333 bp and for B isoforms the amplification product was 469 bp. PCR products were cloned into pCRII vector (Invitrogen). RNA probes (antisense and sense as negative controls) were obtained by in-vitro transcription with T7 RNA polymerase of about 1 ug of linearized plasmid, using a DIG RNA labelling kit from Roche according to the protocol supplied. ISH experiments were performed as previously described [34].

### Methylation analysis

2 μg of genomic DNA extracted from the atrium and ventricle were treated with sodium bisulphite using the methylation gold kit (Zymoresearch). After purification, the DNA was amplified by PCR and cloned within the PGEM-T-easy vector (Promega). The sequences of the oligonucleotides used for amplification are the following: promB forward, AGAGATTTGTAGATTTATTGTTTTT; promB reverse, CAATCATTTATTATACAACTACTACAAC. Twenty independent recombinant clones (ten from atrial DNA and ten from ventricle DNA) were selected, purified and sequenced. The DNA sequence of each recombinant clone was compared to the sequence of the B promoter genomic region reported in the database [GenBank at the NCBI: NG_012232.1]. Conversion of Cs to Ts indicated that Cs were unmethylated in the original DNA, whereas conservation of the Cs indicated that the Cs were methylated.

The studies have been carried following the ethical approval of the local ethical committee (UNIFE) n. 9/2005. Written informed consent for participation in the study was obtained from participants.

## Abbreviations

XLDC: X-Linked dilated cardiomyopathy; DMD: Duchenne muscular dystrophy; BMD: Becker muscular dystrophy; CK: Serum creatine kinase; CCS: Cardiac Conduction System structures.

## Competing interests

The authors declare that they have no competing interests.

## Authors' contributions

MN carried out real-time and in-situ experiments, analysis and interpretation of the data and prepared the manuscript, EV performed the methylation analysis and interpreted the results, GA performed in-situ hybridization and interpreted the results, MB supported Realtime probe design, PS contributed to the patients genomic analysis and interpretation of the results, CR furnished the cardiologic data, FM contributed to the rationale of the experiment and scientifically revised the manuscript, SB contributed to the planning and interpretation of in-situ experiments, GP contributed to the planning and interpretation of the methylation analysis experiments; FG and AF were responsible for the conception and design of the study, interpretation of the results, revision of the manuscript and approval of the final version. All authors read and approved the final manuscript.

## Pre-publication history

The pre-publication history for this paper can be accessed here:

http://www.biomedcentral.com/1471-2350/13/20/prepub
